# The ubiquitylome of developing cortical neurons

**DOI:** 10.17912/micropub.biology.000333

**Published:** 2020-11-28

**Authors:** Shalini Menon, Dennis Goldfarb, Emily M. Cousins, M. Ben Major, Stephanie L. Gupton

**Affiliations:** 1 University of North Carolina at Chapel Hill

**Figure 1. The ubiquitylome of developing cortical neurons:**
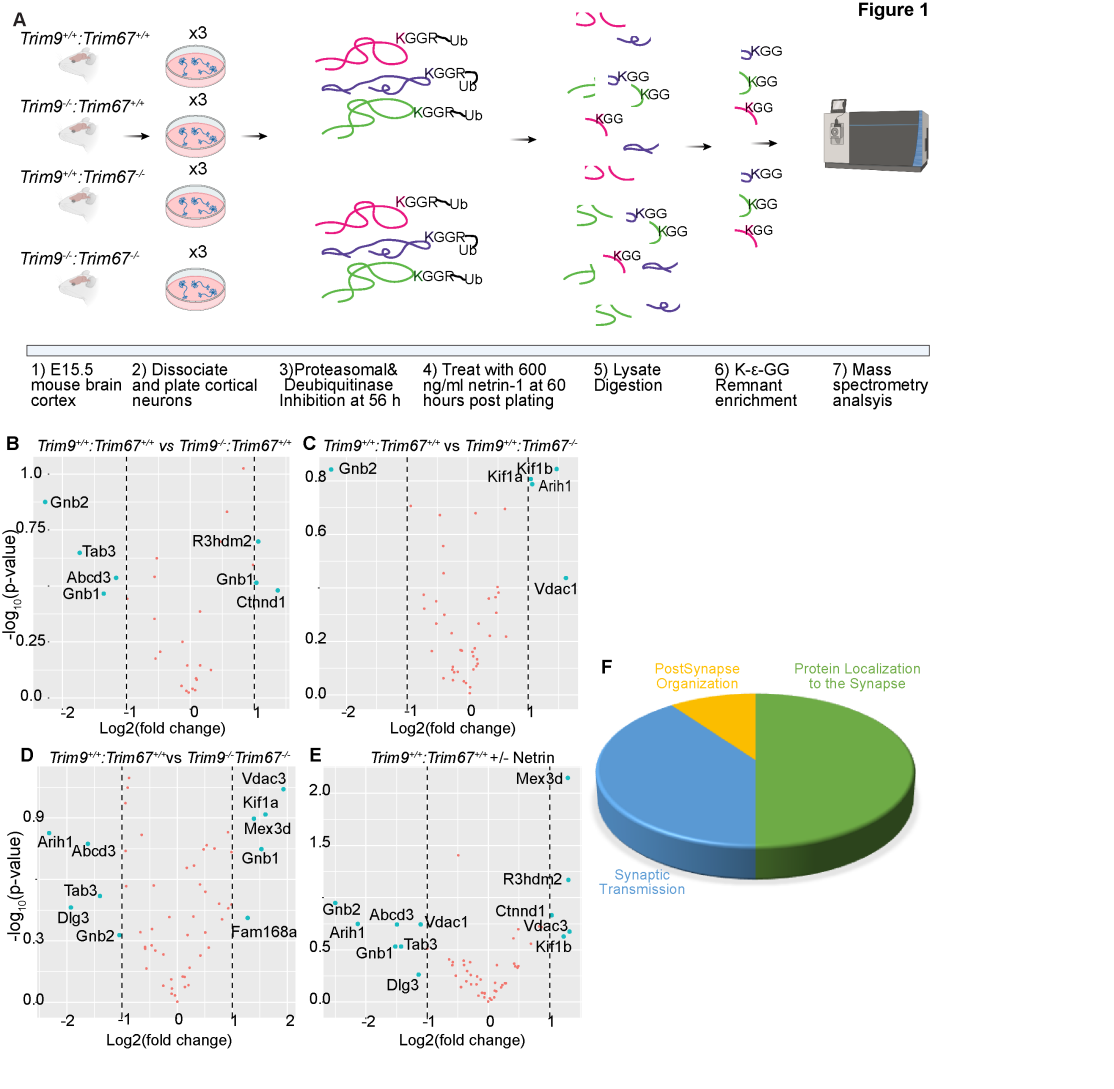
**A)** Graphical representation of approach. Embryonic day 15.5 (E15.5) neurons were dissected from the cortex of mice with indicated genotypes, and cultured for 56 hours prior to addition of proteasomal and deubiquitinase inhibitors for 4 hrs. Half of the plated neurons were treated with 600ng/ml of netrin-1 for 40 min prior to lysis. Neurons were lysed and trypsin digested. Ubiquitylated peptides were enriched and analyzed by mass spectrometry. **B-E)** Volcano plots demonstrating changes in ubiquitylation of proteins that overlapped with high-confidence TRIM9 and TRIM67 interaction partners between the indicated conditions. **F)** Pie-chart of biological processes enriched via Gene Ontology (GO) classification of proteins that overlapped between high-confidence TRIM9 and TRIM67 interaction partners and altered DiGly enrichment.

## Description

Ubiquitylation is a posttranslational protein modification, in which the small protein ubiquitin is covalently attached to substrate proteins. This modification can mark substrates for degradation by the proteasome or lysosomes, or alternatively can alter protein localization, function, trafficking, and protein-protein interactions. Ubiquitylation is a ubiquitous modification, but it is enriched in the brain and often accumulates in neurodegenerative conditions (Fischer *et al.*, 2009).

TRIM9 and TRIM67 are neuronally-enriched E3 ubiquitin ligases (Boyer *et al.*, 2018; Berti *et al.*, 2002). Both genes are required for neuronal morphological responses to the axon guidance cue netrin-1 (Winkle *et al.*, 2014; Boyer *et al.*, 2018; Plooster *et al.*, 2017; Boyer *et al.*, 2020; Menon *et al.*, 2015; Urbina *et al.*, 2018; Winkle *et al.*, 2016). For example, our previously published work demonstrated that the actin polymerase VASP and the netrin receptor DCC exhibit TRIM9 dependent ubiquitylation that is lost upon netrin stimulation (Menon *et al.*, 2015; Plooster *et al.*, 2017; Boyer *et al.*, 2020). Deletion of either gene in the mouseresults in subtle neuroanatomical anomalies yet overt deficits in spatial learning and memory (Menon *et al.*, 2015; Winkle *et al.*, 2016; Boyer *et al.*, 2018). Despite their role in neuronal form and function, the identity of few TRIM9 or TRIM67 substrates are known. Here we performed ubiquitin remnant profiling approach (Xu *et al.*, 2010) in cultured wildtype and knockout murine embryonic cortical neurons to identify ubiquitylated peptides and proteins, with the ultimate goal of identifying substrates of TRIM9 and TRIM67 that exhibited reduced ubiquitylation in the absence of the ligase. To identify substrates that show either netrin-dependent or netrin-sensitive ubiquitination, experiments were done with or without supplementation of cultures with the guidance cue netrin-1.

Ubiquitylated peptides were enriched from cultured cortical neurons from *Trim9^+/+^*/*Trim67^+/+^*, *Trim9^-/-^*/*Trim67^+/+^*, *Trim9^+/+^*/*Trim67^-/-^*, *Trim9^-/-^*/*Trim67^-/-^*embryos at 2.5 days in vitro (DIV) with or without 40 minutes of netrin-1 supplementation (**Figure 1A)**. The enriched peptides were analyzed by LC/MS/MS. When compiled across all four genotypes and netrin treatment in triplicate, this generated a list of 854 unique proteins and 1454 unique peptides that were ubiquitylated during early neuronal development (Table 1 See Extended Data). The data described in Table 1, sheet 2 include gene names of identified proteins with NCBI identifiers; the peptide sequences identified by MS; the peptides with ubiquiylated lysine; the number of glycine adducts per peptide; the types of samples (genotype and netrin treatment) and replicates; and median subtracted intensity before and after batch correction. Although no differences in ubiquitylation between any condition reached statistical significance, likely due to batch variability between replicates and insufficient sample size, these results revealed the vast array of protein ubiquitylation during this developmental window.

In a previous study, we identified a putative TRIM9 and TRIM67 interactome using proximity-dependent labeling with the promiscuous biotin ligase (BirA*) attached to either TRIM9 or TRIM67 (Menon *et al.*, 2020). These interactions were ranked by likelihood of interaction based on SAINT’s interaction probability and a false discovery rate (FDR) threshold that was assigned to each of the identified proteins. A comparison of the ubiquitylated proteome from developing brains with the top 25% of the interaction candidates identified peptides from 55 proteins that showed an increase or decrease (albeit insignificant) in ubiquitylation levels in the absence of *Trim9,* *Trim67,* or both relative to wildtype (Figure 1B-D). Similar analysis revealed proteins with altered ubiquitylation upon netrin application. Gene ontology analysis of these 55 proteins demonstrated synaptic enrichment, with only three categories identified: protein localization to the synapse, synaptic transmission, and postsynapse organization. This is remarkable as both proteomic approaches were done in cortical neurons well before synapses formed. However a synaptic function for TRIM9 and TRIM67 is consistent with behavioral phenotypes identified in knockout animals (Winkle *et al.*, 2016; Boyer *et al.*, 2018b). Six and five proteins were identified as candidate TRIM9 and TRIM67 substrates, respectively. Proteins that exhibited differential ubiquitylation upon loss of *Trim9* included Abcd3, Ctnnd1, Gnb1, Gnb2, R3hdm2, Tab3. Proteins that exhibited differential ubiquitylation upon loss of *Trim67* included Arih1, Gnb2, Kif1a, Kif1b, Vdac1. Similarly, Abcd3, Arih1, Dlg3, Gnb1, Gnb2, Kif1a, Mex3d, Tab3, Tcrp1, Vdac3 were found to be differentially ubiquitylated in *Trim9^-/-^*:*Trim67^-/-^* neurons relative to *Trim9^+/+^:Trim67^+/+^* neurons. Many of these proteins contained multiple peptides that were ubiquitylated. For instance, three different peptides were mapped to Gnb2, of which one peptide (LLLAGYDDFNCNIWDAMub(K)GDR) showed a consistent, although insignificant decrease in ubiquitylation across all three genotypes compared to the wildtype. Similarly, of the multiple peptides identified for Gnb1, ub(K)ACADATLSQITNNIDPVGR and LLLAGYDDFNCNVWDALub(K)ADR showed opposite trends in ubiquitylation in the absence of *Trim9*. Although previous work describe nonredundant functions of TRIM9 and TRIM67, the increased of ubiquitination of Vdac3, Mex3d, and FAM168a and decreased ubiquitintation of Dlg3, Tab3, only in *Trim9^-/-^*:*Trim67^-/-^* but not single knockout neurons may suggest shared substrates or redundant functions of TRIM9 and TRIM67. Further exploration will be needed to validate TRIM9 and TRIM67 dependent ubiquitylation of these proteins during neuronal development. The identification of ubiquitylated peptides and proteins in developing cortical neurons will be illuminating for many future studies.

## Methods

**Animals**

All mouse lines were on a pure inbred C57BL/6J background and bred at UNC with approval from the Institutional Animal Care and Use Committee. Timed pregnant females were obtained by placing male and female mice together overnight; the following day was designated as E0.5 if the female had a vaginal plug. Generation of *Trim9^-/-^* mice (MGI ID:5660333, Trim9^tm1.1Slgu^) and *Trim67^-/- ^*mice (MGI ID:6259600, Trim67^96^) have been described previously (Boyer *et al.*, 2018; Winkle *et al.*, 2014).

**Cortical neuron culture**

E15.5 litters were removed from wildtype, *Trim9^-/-^, Trim67^-/- ^*and *Trim9^-/-^:Trim67^-/-^* pregnant dams by postmortem cesarean section. Dissociated cortical neuron cultures were prepared as described previously (Kwiatkowski *et al.*, 2007). Briefly, cortices were micro-dissected, cortical neurons were dissociated with 0.25% trypsin for 20 min at 37°C, followed by quenching of trypsin with Trypsin Quenching Medium (TQM, neurobasal medium supplemented with 10% FBS and 0.5 mM glutamine). After quenching, cortices were gently triturated in TQM. Dissociated neurons were then pelleted at 100 g for 7 min. The pelleted cells were gently resuspended in Serum Free Medium (SFM, neurobasal medium supplemented with B27 (Invitrogen) and plated on tissue culture plastic coated with 1 mg/ml poly-D-lysine (Sigma-Aldrich).

**Enrichment of ubiquintated peptides**

Primary cortical neurons from *Trim9^+/+^*/*Trim67^+/+^* (wildtype,WT), *Trim9^-/-^*/*Trim67^+/+^* (*Trim9* null), *Trim9^+/+^*/*Trim67^-/-^* (*Trim67* null), *Trim9^-/-^*/*Trim67^-/-^*(*Trim9*/*Trim67* double knockout, 2KO) murine E15.5 litters were dissociated and cultured on Poly-D-Lysine (Sigma) treated dishes. Approximately 56 hrs post-plating, cells were treated with 100 nM Bortezomib and 5 µM PR-619 for 4 hrs. 40 min prior to collecting cells, half of all plated cells were treated with 600 ng/ml of netrin-1. Cells were harvested and stored at -80°C until dissociated neurons from 20-25 pups per genotype per condition were collected. The harvested cells were lysed for 30 min on ice in 1000 µl of freshly prepared buffer (8 M urea in 50 mM Tris-HCl pH7.5, 150 mM NaCl, 1 mM EDTA, 2 µg/ml apoprotinin, 10 µg/ml leupeptin, 1 mM PMSF, 50 µM PR-619 and 1 mM Iodoacetamide). Lysates were sonicated 3x 30 s cycle at 10% output, and clarified at 21.1k g at 4˚C for 15 min. Protein concentration was estimated using the Bio-Rad Protein Assay Dye Reagent. 5 mg of clarified lysates were then reduced with 5 mM DTT at room temperature for 45 min followed by alkylation for 30 min at room temperature using 10 mM chloroacetamide. To avoid the possibility of overalkylation we used chloroacetamide instead of iodoacetamide (Nielsen *et al.*, 2008). The reduced and alkylated protein lysates were diluted using 50 mM Tris-Cl to obtain a final concentration of 2 M urea in solution followed by overnight digestion with trypsin (1:1000)(Promega). Pre- and post- trypsinization samples were resolved on a 4-20% gradient gel (Mini-PROTEAN TGX Stain-Free precast gel (Bio-Rad)) to confirm efficiency of trypsin digestion. Pre-digested samples showed multiple bands along the entire length of the gel and the digested samples showed a thick band at the bottom of the gel. Peptides were then desalted using C18 Sep-Pack columns (Waters). Eluted peptides are lyophilized following which immunoaffinity purification was performed using the PTMScan Ubiquitin Remnant Motif (K-GG) Kit (Cell Signaling Technologies). The affinity purified peptides were desalted using Pierce C18 columns (Thermofisher).

**Mass spectrometry and data analysis**

Reverse-phase nano-high-performance liquid chromatography (nano-HPLC) coupled with a nanoACQUITY ultraperformance liquid chromatography (UPLC) system (Waters Corporation; Milford, MA) was used to separate trypsinized peptides. Trapping and separation of peptides were performed in a 2 cm column (Pepmap 100; 3-m particle size and 100-Å pore size), and a 25-cm EASYspray analytical column (75-m inside diameter [i.d.], 2.0-m C18 particle size, and 100-Å pore size) at 300 nL/min and 35°C, respectively. Analysis of a 150-min. gradient of 2% to 25% buffer B (0.1% formic acid in acetonitrile) was performed on an Orbitrap Fusion Lumos mass spectrometer (Thermo Scientific). The ion source was operated at 2.4kV and the ion transfer tube was set to 300°C. Full MS scans (350-2000 m/z) were analyzed in the Orbitrap at a resolution of 120,000 and 1e6 AGC target. The MS2 spectra were collected using a 1.6 m/z isolation width and were analyzed either by the Orbitrap or the linear ion trap depending on peak charge and intensity using a 3 s TopSpeed CHOPIN method. Orbitrap MS2 scans were acquired at 7500 resolution, with a 5e4 AGC, and 22 ms maximum injection time after HCD fragmentation with a normalized energy of 30%. Rapid linear ion trap MS2 scans were acquired using an 4e3 AGC, 250 ms maximum injection time after CID 30 fragmentation. Precursor ions were chosen based on intensity thresholds (>1e3) from the full scan as well as on charge states (2-7) with a 30-s dynamic exclusion window. Polysiloxane 371.10124 was used as the lock mass.

For the data analysis all raw mass spectrometry data were searched using MaxQuant version 1.5.7.4. Search parameters were as follows: UniProtKB/Swiss-Prot mouse canonical sequence database (downloaded 1 Feb 2017), static carbamidomethyl cysteine modification, specific trypsin digestion with up to two missed cleavages, variable protein N-terminal acetylation (+42.011) and methionine oxidation (+15.995), digly variable modification on lysines (+114.043), match between runs (0.7min match time window and 30min alignment time window), and label-free quantification (LFQ) with a minimum ratio count of 2. Protein and peptide identifications were each filtered for a false discovery rate of 1% determined by a target/decoy search strategy that used reversed peptide sequences as decoys. All unmentioned parameters were kept at their defaults.

For downstream analysis, common contaminants, reverses, and non-digly peptides were removed. Digly peptide intensities were then log2 transformed and missing values were imputed from a normal distribution using Perseus (Tyanova *et al.*, 2016) with width = 0.3 and down shift = 1.8. All intensities were then median normalized per experiment by subtracting an experiment’s median log2 intensity from all the peptides in that experiment. Finally, batch correction was performed separately for each peptide using the *removeBatchEffect* function from the LIMMA package (Ritchie *et al.*, 2015) by treating the three sets of replicates as separate batches. The mass spectrometry proteomics data including raw intensities pre-normalization have been deposited to the ProteomeXchange Consortium via the PRIDE (Perez-Riverol *et al.*, 2019) partner repository with the dataset identifier PXD021818.

**Data representation and Gene Ontology (GO) analysis**

Gene ontology enrichment analysis of the neuronal ubiquitinome was performed using the ClueGo application available for download in the Cytoscape_3.7.
